# Prognostic and clinicopathological significance of TLR4 expression in patients with breast cancer: a meta-analysis

**DOI:** 10.3389/fonc.2024.1344130

**Published:** 2024-02-23

**Authors:** Jinxu Wen, Jiayi Zhang, Xiaoyong Wu, Xuemin Yan, Xiaoru Qin, Yuexin Wang

**Affiliations:** ^1^ Department of Thyroid and Breast Surgery, Hebei General Hospital Affiliated to Hebei Medicine University, Shijiazhuang, Hebei, China; ^2^ Department of Thyroid and Breast Surgery, Hebei General Hospital Affiliated to North China University of Science and Technology, Shijiazhuang, Hebei, China; ^3^ Department of Thyroid and Breast Surgery, Hebei General Hospital, Shijiazhuang, Hebei, China

**Keywords:** breast cancer, toll-like receptors, toll-like receptor 4, TLR4, prognosis

## Abstract

**Background:**

The prognostic value of Toll-like receptor 4 (TLR4) in breast cancer remains to be determined. Therefore, this paper aims to conduct a meta-analysis to assess the correlation between TLR4 and clinicopathological indicators as well as survival outcomes in breast cancer.

**Method:**

Related literature retrieved from Embase, PubMed, Cochrane Library, Web of Science, China National Knowledge Infrastructure (CNKI) and China Wanfang. The search deadline is April 12, 2023. The outcome measures employed in the study comprised hazard ratio (HR), odds ratio (OR), and 95% confidence interval (CI) as effective indices. The data analysis was conducted using Stata 17.0 software.

**Results:**

High TLR4 expression was associated with lymph node metastasis (OR=2.077, 95%CI=1.160-3.717, *P*= 0.014), tumor size (≥2 cm) (OR=2.194, 95%CI= 1.398-3.445, *P*= 0.001), PR expression (OR = 0.700, 95% CI = 0.505–0.971, *P*= 0.033), and clinical stage (OR = 3.578, 95%CI= 3.578-5.817, *P*<0.05), but not with histological grade (95%CI= 0.976-1.735, *P*= 0.072), ER expression (OR = 1.125, 95% CI = 0.492–2.571,*P*= 0.781), and HER-2 status (OR = 1.241, 95% CI = 0.733–2.101, *P* = 0.422). In addition, TLR4 overexpression was an independent prognostic indicator of DFS (HR= 1.480, 95%CI= 1.028- 2.130, *p*= 0.035) in breast cancer patients, but not related to OS(HR=1.730, 95%CI= 0.979-3.057, *P*= 0.059).

**Conclusions:**

From our main analysis results, high TLR4 expression is associated with lymph node metastasis, larger tumor size (≥2 cm), later clinical stage, negative PR expression and shorter DFS, suggesting poor prognosis in breast cancer patients.

## 1 Introduction

Breast cancer (BC) is the most prevalent malignant neoplasm among women worldwide. According to statistics, breast cancer has surpassed lung cancer to become the leading cause of cancer worldwide (1). Breast cancer is a heterogeneous disease with diverse molecular subtypes, which can be categorized into five distinct groups based on the expression of estrogen receptor (ER), progesterone receptor (PR), human epidermal growth factor receptor 2 (HER-2) and nuclear protein Ki-67 (2, 3). In patients with breast cancer, the outcome of prediction and treatment response depends on immunohistochemical (IHC) markers such as ER, PR, and HER-2, as well as standard clinicopathologic features such as tumor size, grade, and lymph node involvement, which often exhibit potentially different outcomes due to other clinical manifestations and molecular characteristics. Although such patients can be treated by surgery, endocrine therapy, radiotherapy, chemotherapy and targeted therapy, there is still a lack of effective treatment options for patients with advanced stage and metastasis. In the future, more rational treatment options are expected to be provided through experimental and clinical studies on effective prognostic biomarkers.

Toll-like receptors (TLRs) are known to be type I trans-membrane glycoproteins and members of the TLR-IL-1 superfamily, were first discovered in fruit flies, mostly expressed in cells of the innate immune system (macrophages and dendritic cells (DCs)), play an indispensable role in activation/inhibition of immune and non-immune cells via recognition of pathogen-associated molecular patterns (PAMPs) and damage-associated molecular patterns (DAMPs) (4, 5). In recent years, many results have revealed the function and molecular mechanisms of TLRs in cancer, suggesting that TLRs may play a role in the development of cancer (6). Among them, TLR4 has been widely studied as one of the essential members of the TLR family, which plays a role in promoting many inflammatory diseases. More and more evidence shows that the abnormal expression of this receptor in tumor cells and tumor microenvironment of various cancer types is highly correlated with the initiation of tumourigenesis, tumor progression and drug resistance (7).

TLR4 is overexpressed in the majority of clinical breast cancer samples and 68% of the examined BC lines (8). Related literature reports suggest that TLR 4 is a key molecule involved in breast cancer cell eradication or induction of breast cancer development and normal cell transformation, in normal breast tissue, TLR4 fights breast cancer cells by recognizing their DAMPs, but overexpression of TLR4 and changes in its signaling pathway are vital factors that alter the function of TLR4 against breast cancer and promote the development and metastasis of breast cancer (5). Although some studies have reported the relationship between TLR4 and clinicopathologic features and survival indicators of breast cancer, their conclusions are inconsistent. Therefore, meta-analysis is the most effective method to deeply understand the impact of TLR4 on clinicopathologic features and prognosis of breast cancer patients.

## 2 Materials and methods

### 2.1 Search strategy

The following databases were searched for the retrieval of relevant data: Embase, PubMed, Cochrane Library, Web of Science, China National Knowledge Infrastructure (CNKI) and China Wanfang. The search deadline is April 12, 2023. The key terms used in the searches included”Toll-Like Receptor 4”;”TLR4”;”Breast Neoplasms”;”Breast tumors”;”Breast Cancer”;”Malignant Breast Neoplasm”, at the same time combined with the free words and the subject words of each database, the use of logical characters, wildcards and range operators to develop the search mode.

### 2.2 Inclusion and exclusion criteria

The inclusion criteria for the relationship between TLR4 and breast cancer in this meta-analysis are as follows: (1) Research must be original and published; (2) All observed patients must be pathologically diagnosed with breast cancer and have reported associations between TLR4 and clinicopathologic features of breast cancer and/or overall survival (OS) and/or disease-free survival (DFS); (3) The expression of TLR4 in tumor tissue was detected. Exclusion criteria include: (1) Letters, meetings, abstracts, reviews or case reports; (2) Research reports in languages other than Chinese and English; (3) Non-human breast cancer research; (4) Studies lacking data on assessing hazard ratio (HR), odds ratio (OR), and 95% confidence interval (95%CI); (5) Studies have duplicate data or analysis.

### 2.3 Data extraction and quality assessment

**Figure 1:**
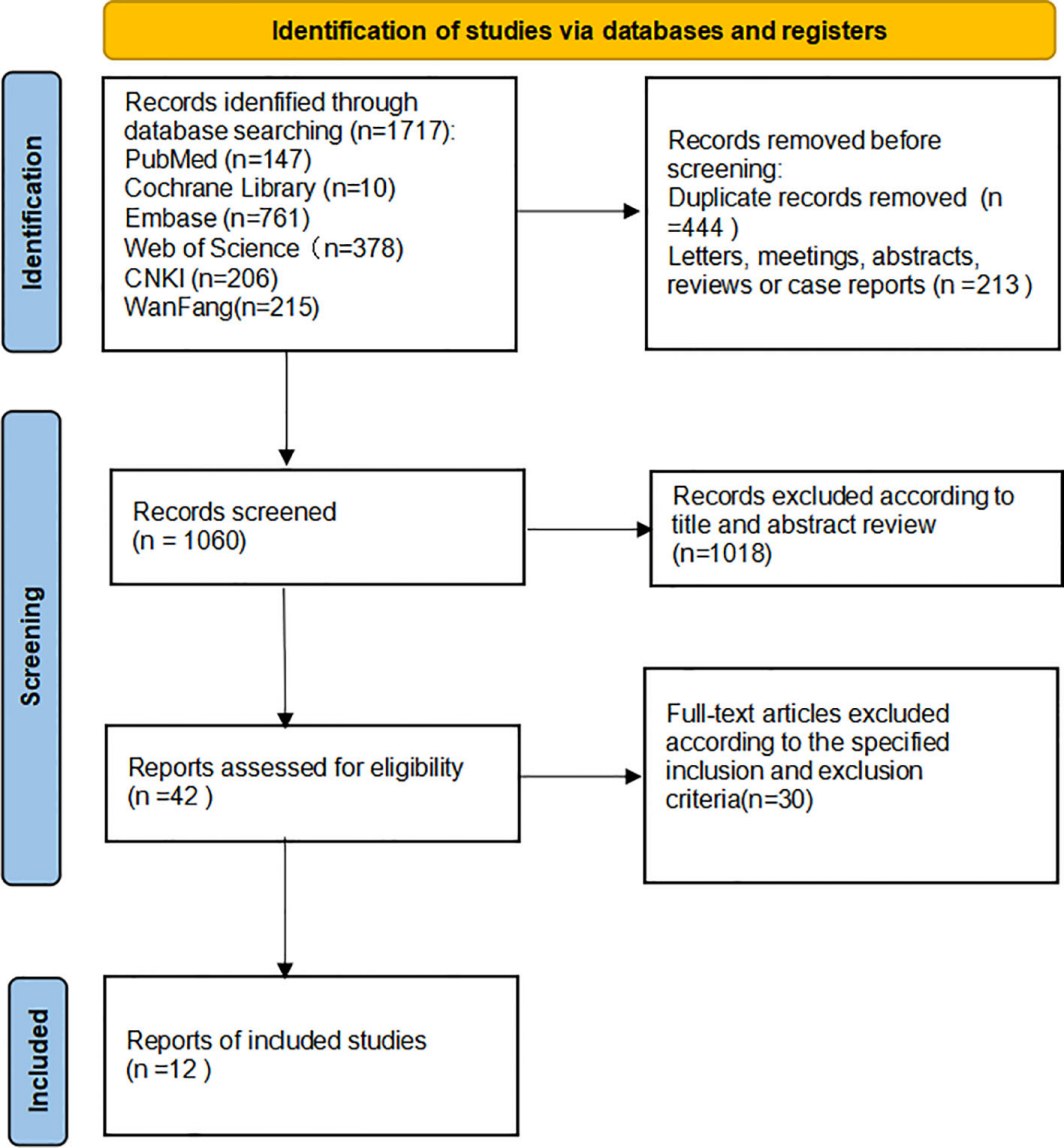
The entire process of literature search has been depicted in the flow diagram.

The studies were reviewed and screened by two researchers, independently obtaining data from the available studies. If the research retrieved cannot be classified by title and abstract, the full text is reviewed. The two investigators negotiated with each other and reached a consensus by soliciting the opinions of the third investigator in any differences, in the specific literature search and deletion process, as shown in **Figure 1**. During the data review, and the following details were recorded: First author, year of publication, nationality, experimental method, sample size, p(lymph node metastasis, tumor size, clinical stage, histological grade), outcome indicators involving multivariate analysis, and Newcastle-Ottawa Scale (NOS) score (population selection, comparability score, and results score) in **Table 1**. Quality analysis of each extracted study was conducted by two independent researchers using NOS scores, and the consensus was reached through negotiation with a third party when there were different opinions. A total of twelve studies with scores ranging from 6 to 9 were included for analysis (9–14, 16–20). Exposure factors were measured by two researchers based on selection criteria for the study population, independent semi-quantitative evaluation of the selected studies, comparability with other studies, and the use of NOS quality reviews. The results were mainly divided into: good quality (7-9), medium quality (6-7), poor quality (≤5).

**Table 1:** The basic characteristics of the enrolled papers in the study.

Author	Year	Country	Method	Number	P							Follow-up (months)	DFS	OS	NOS
					LNM	Stage	TSi	Grade	ER	PR	HER-2				
Zhe,S (9).	2022	China	IHC	50	<0.05	0.05	0.01	0.05	NR	NR	NR	NR	NR	NR	7
Saponaro, C (10).	2021	Italy	IHC	374	0.07	NR	NR	0.06	0.02	0.11	0.05	67	0.01	0.04	9
Xueqiong,X (11).	2019	China	IHC	100	0.04	0.02	0.02	NR	NR	NR	NR	NR	NR	NR	7
Xuebo, W (12).	2017	China	IHC	120	0.02	0.01	0.01	0.02	0.01	0.11	0.17	NR	NR	NR	7
Fangjing,M (13).	2017	China	IHC	200	NR	NR	NR	NR	NR	NR	NR	102	0.59	0.63	6
Chen, X.J (14).	2015	China	IHC	60	<0.05	0.01	NR	0.34	NR	NR	NR	NR	NR	NR	7
Mehmeti, M (15).	2015	Sweden	IHC	128	NR	NR	NR	NR	NR	NR	<0.05	NR	NR	NR	7
Fangjing,M (16).	2014	China	IHC	205	0.20	NR	NR	0.34	0.06	0.02	0.15	98	0.63	0.63	8
Wenjun,W (17).	2014	China	IHC	58	0.03	0.04	0.74	0.56	NR	NR	NR	NR	NR	NR	7
Ehsan,N (18).	2013	Pakistan	IHC	50	0.02	NR	NR	0.50	NR	NR	NR	NR	NR	NR	7
B. Petricevic (19).	2012	Croatia	IHC	133	0.61	NR	0.29	NR	0.13	0.57	0.69	60	NR	NR	8
Baojun,L (20).	2011	China	IHC	106	<0.05	<0.05	NR	>0.05	NR	NR	NR	NR	NR	NR	6

LNM, lymph node metastasis; TSi, tumor size; ER, estrogen receptor; PR, progesterone receptor; HER-2, human epidermal growth factor receptor-2; NR, not report in literature; DFS, disease-free survival; OS, overall survival.

### 2.4 Statistical analysis

We used STATA 17.0 to analyze the statistical data, HR, OR, and 95%CI were used as the statistics of effect analysis to evaluate the expression characteristics and clinical significance of TLR4 in breast cancer. Cochran’s Q-test and Higgins I^2^ statistic were performed to quantify the degree of heterogeneity among the selected studies, When P≥0.1 and I^2^ ≤50%, it indicated no heterogeneity or small heterogeneity among the study results. The fixed-effect model combined the effect size; otherwise, the random-effect model was used for data analysis. The assessment of publication bias was performed by using the Begg and Egge Test. Meanwhile, a sensitivity analysis was performed to discuss the source of heterogeneity and the magnitude of its contribution. *p <*0.05 was considered to be statistically significant.

## 3 Results

### 3.1 Identifification of relevant studies

A total of 1717 potentially relevant studies were selected through a preliminary search. Among them, 657 articles were deleted because of duplicate records, letters, meetings, abstracts, reviews, and case reports. Secondly, 1080 articles were excluded by reading the title and abstract of the article. Finally, after in-depth reading of the complete text, twelve articles were selected for meta-analysis according to the specified inclusion and exclusion criteria (9–20). The flowchart depicts the entire process involved in retrieving literature (**Figure 1**).

### 3.2 Study characteristics

A total of 1584 patients with breast cancer were included in this meta-analysis. Of all the included studies, ten, five, six, eight, four, four and five reported data on TLR4 in lymph node metastasis, tumor size, clinical stage, histologic grade, ER, PR and HER-2 respectively and three studies reported data on the association of TLR4 with survival outcomes. The correlation between TLR4 and clinicopathologic features and prognosis of breast cancer patients is shown in **Table 1**.

### 3.3 TLR4 expression is associated with breast cancer progression

**Figure 2:**
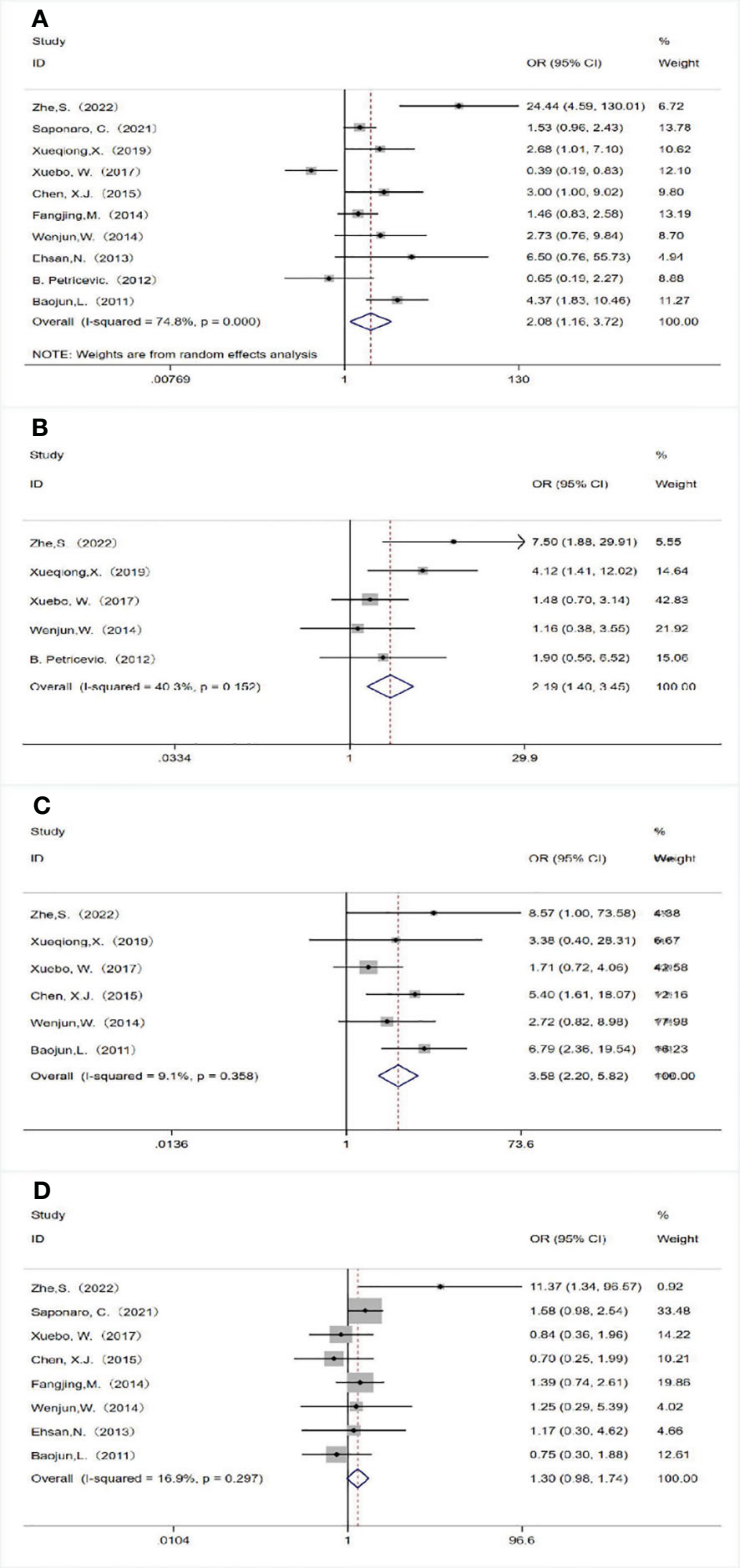
Association between TLR 4 expression and the clinicopathological features of breast cancer. **(A)** Lymph node metastasis; **(B)** Tumor size; **(C)** Clinical stage; **(D)** Histological grade.

A total of 10 studies analyzed the relationship between TLR4 expression and lymph node metastasis. Due to the heterogeneity of study results (*P<*0.05, I^2 ^= 74.8%), we chose the random effects model for analysis, and the combined OR was 2.077 (95%CI=1.160-3.717, *P*= 0.014; **Figure 2A**). This indicated that high expression of TLR4 was significantly correlated with the presence of lymph node metastasis. Five studies observed the relationship between TLR4 expression and tumor size, and we selected the fixed-effect model for analysis. Combined results showed that high TLR4 expression was significantly associated with larger tumor size (≥2 cm)(OR=2.194, 95%CI= 1.398-3.445, *P*= 0.001; **Figure 2B**),there was no heterogeneity in the results(*P* =0.152, I^2 ^= 40.3%).We analyzed six studies using the fixed-effect model and found that high TLR 4 expression was associated with a more advanced clinical stage (OR = 3.578, 95%CI= 3.578-5.817, *P<*0.05; **Figure 2C**) with no heterogeneity (*P* =0.358, I^2 ^= 9.1%). Eight studies examined the relationship between TLR 4 expression and histological grade, due to insignificant heterogeneity of findings (*P* = 0.297, I^2 ^= 16.9%), the OR value obtained by fixed effect model is 1.302 (95%CI= 0.976-1.735, *P* = 0.072; **Figure 2D**), results showed that TLR 4 expression was not associated with histological grade.

### 3.4 Associations between TLR 4 expression and molecular subtype of breast cancer

**Figure 3:**
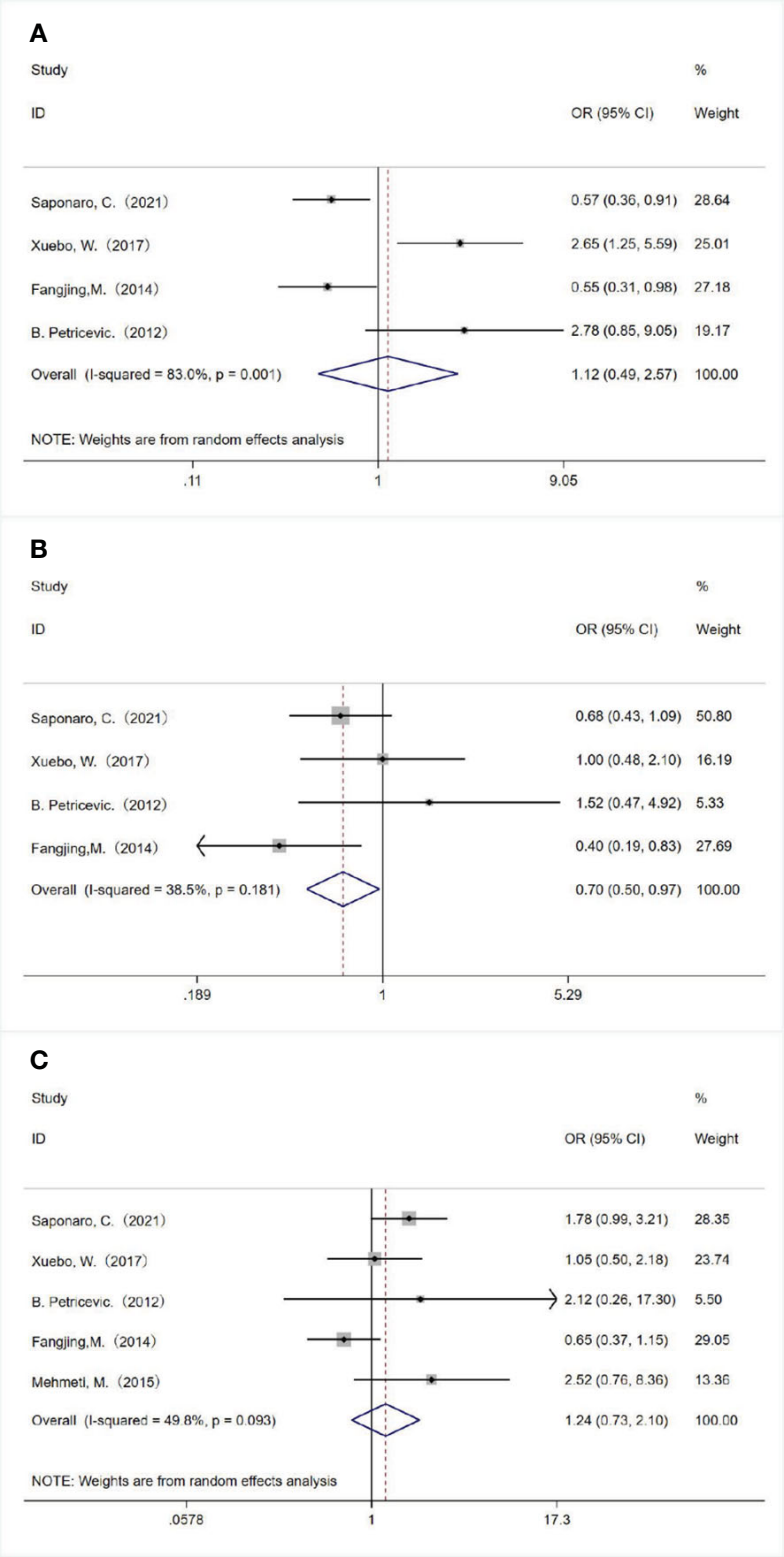
Associations between TLR 4 expression and molecular subtype of breast cancer. The relationships between TLR 4 expression and **(A)** estrogen receptor status, **(B)** progesterone receptor status and **(C)** human epidermal growth factor receptor 2 status.

We also analyzed the associations between TLR4 expression and the molecular subtype of breast cancer. TLR4 overexpression was significantly associated with negative progesterone receptor (PR) expression (OR = 0.700, 95% CI = 0.505–0.971, *P* = 0.033 using a fixed-effect model; **Figure 3B**). There was no significant relationship between TLR4 overexpression with estrogen receptor (ER) expression (OR = 1.125, 95% CI = 0.492–2.571, *P* = 0.781 using a random-effect model; **Figure 3A**) and human epidermal growth factor receptor 2 (HER2) status (OR = 1.241, 95% CI = 0.733–2.101, *P* = 0.422 using a random-effect model; **Figure 3C**).

### 3.5 Relationship between TLR4 expression and survival outcomes in patients with breast cancer

**Figure 4:**
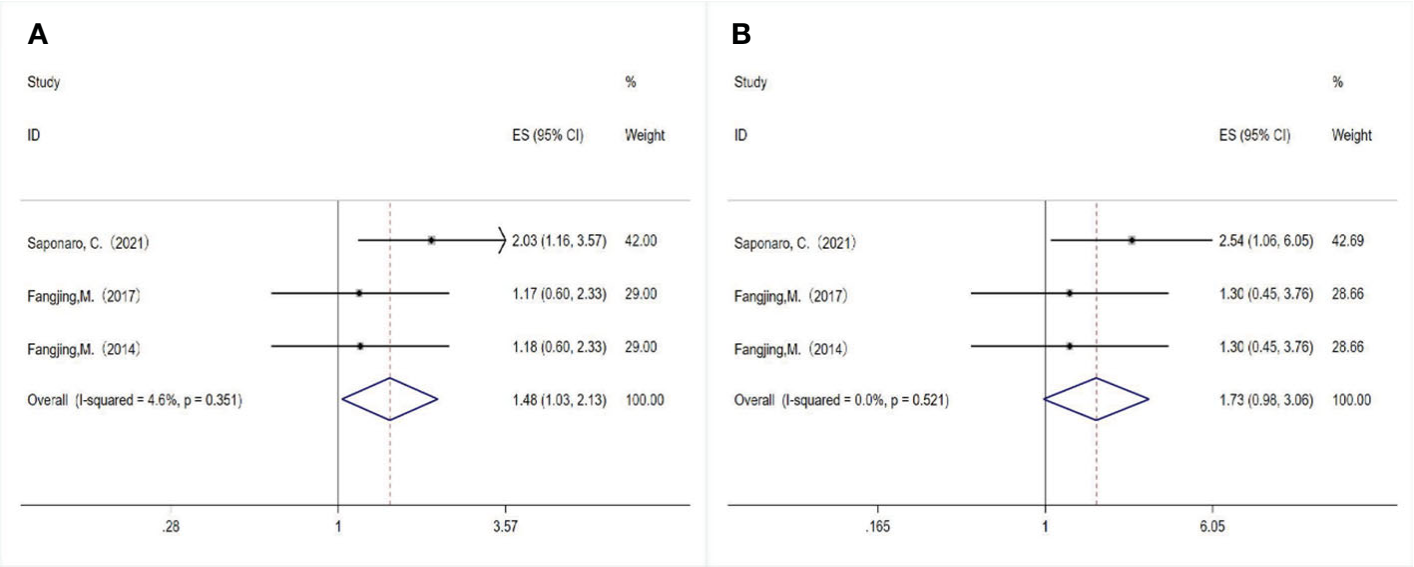
Relationship between TLR4 expression and survival outcomes in breast cancer patients. **(A)** Disease-free survival; **(B)** Overall survival.

A total of three studies examined the association between TLR4 expression and DFS/OS. Although two studies had the same first author and the sample selection time overlapped, the samples were from different databases, so we included these studies and evaluated the relationship between TLR4 expression and survival outcomes in breast cancer patients. We used the fixed-effect model (*P*= 0.351, I ^2 ^= 4.6%) combined with HR (HR= 1.480, 95%CI= 1.028- 2.130, *P*= 0.035; **Figure 4A**), indicating that overexpression of TLR4 was significantly correlated with shortening of DFS. In addition, we evaluated the relationship between TLR4 and OS using a fixed-effect model, and the combined HR was 1.730 (95%CI= 0.979-3.057, *P*= 0.059; **Figure 4B**), the results showed no heterogeneity (*P*=0.521, I^2 ^= 0.0%). The results showed that TLR4 expression was not correlated with OS.

### 3.6 Sensitivity analysis

**Figure 5:**
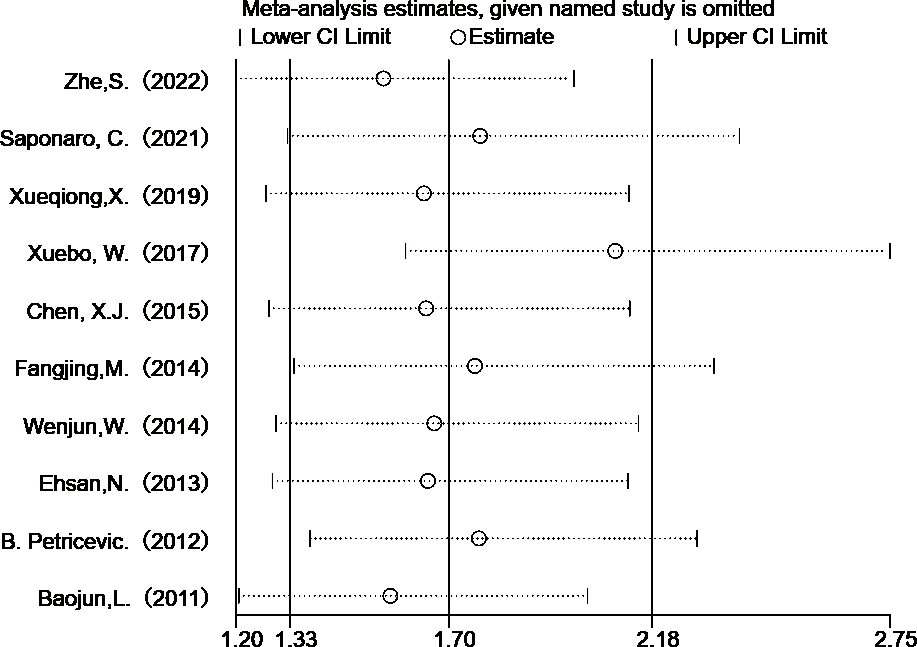
Sensitivity analysis of TLR 4 expression in lymph node metastasis studies in breast cancer patients.

Heterogeneity exists in the included studies on lymph node metastasis. Through sensitivity analysis, we found that Wang’s study was compassionate (**Figure 5**). After excluding Wang’s study, we further analyzed the remaining nine studies by using the fixed effects model method, and the OR value was 2.097 (95%CI= 1.599-2.749), *P<*0.05), the results were still heterogeneous (*P*= 0.012, I^2 ^= 59.2%). Subsequently, we excluded the study with the smallest proportion and obtained an OR value of 1.899 (95%CI= 1.437-2.509, *P<*0.05), and there was no heterogeneity (*P*= 0.152, I^2 ^= 34.6%). This shows that removing the study of Wang and Shi can eliminate the heterogeneity of this analysis. Although the study of Wang and Shi was the source of heterogeneity, it did not change the combined results, indicating that the results of this analysis are stable and reliable.

### 3.7 Published bias analysis

**Figure 6:**
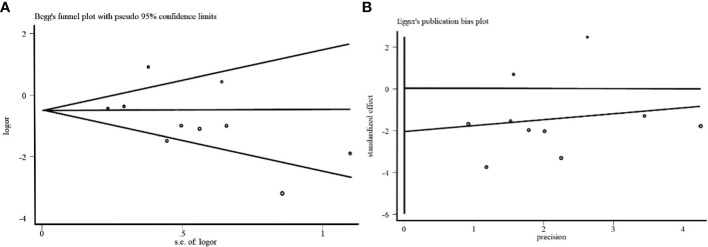
**(A)** Begg funnel plot of the effect of TLR 4 expression on lymph node metastasis in breast cancer. **(B)** Egger funnel plot of the effect of TLR 4 expression on lymph node metastasis in breast cancer.

To evaluate the stability of the overall estimate, We used the Begg funnel plot and the Egger linear regression test to examine the ten studies reporting the effect of TLR 4 expression on lymph node metastasis. No publication bias was detected in the Begg test (*p*> | Z | =0.210; **Figure 6A**) and the Egger test (*p*> | t | =0.193; **Figure 6B**).

## 4 Discussion

TLRs play a role in the pathogenesis of cancer, where TLR is expressed on tumor cells, and dying tumor cells release endogenous TLR ligands, which activate the TLR signaling pathway and promote tumourigenesis (21). Activation of TLR4 occurs via binding with its ligand lipopolysaccharide (LPS), which is a constituent of the outer membrane in Gram-negative bacteria. Upon LPS binding, TLR4 dimerizes and recruits downstream signaling and/or adapter molecules, TLR4 can recruit four adapters, such as myeloid differentiation Factor 88, TIRAP, TRIF, and TRAM, leading to the expression of genes associated with cancer cell proliferation, survival, invasion, and metastasis (22, 23). The mechanisms by which TLR4 promotes metastasis include the facilitation of an epithelial-mesenchymal transition (EMT) and the elevation of cytokines (IL-6, IL-10) that boost matrix metalloproteinase (MMP2 and MMP9) expression (24). In laryngeal cancer, KIF26B antisense RNA 1 regulates TLR4 and activates the TLR4 signaling pathway to promote malignant progression (25). In ovarian cancer, LPS induced the activation of TLR4, up-regulated osteopontin, and increased the malignant phenotype of ovarian cancer cells (26). In hepatocellular carcinoma, TLR4 induces human hepatocellular carcinoma through a variety of mechanisms, including increased production of pro-inflammatory and malignant-related molecules (27). In addition, TLR4 is also associated with the proliferation and invasion of malignant tumors such as colorectal cancer (28)and prostate cancer (29).

TLR4 is highly expressed in a variety of malignant tumor cells and is associated with clinicopathological features and prognostic indicators. TLR4 was expressed at high levels in cervical cancer cells and was associated with the clinical FIGO stage and lymph node metastasis (30). It is highly expressed in papillary thyroid carcinoma tissue and is associated with lymph node metastasis and advanced TNM stage (31); High expression in lung cancer cells is significantly correlated with histological type, clinical TNM staging, and the presence of lymphatic infiltration, and suggests a poor prognosis of non-small cell lung cancer (32). Although the relationship between TLR4 and survival prognosis of various cancers has been reported, and it believes that elevated TLR4 expression is associated with poorer OS and shorter DFS in patients with solid tumors (33), until now, there has been no systematic study on the relationship between TLR4 and breast cancer.

A total of twelve kinds of literature were included for meta-analysis. From the perspective of the relationship between TLR4 expression and clinicopathological features, a total of ten studies, including 1175 breast cancer patients, analyzed the relationship between TLR4 expression and lymph node metastasis. Although the results were heterogeneous, the heterogeneity could be significantly reduced by sensitivity analysis and eliminating the studies with the most minor proportion. Moreover, the combined results did not change, indicating that our results were stable and reliable, and high expression of TLR4 could predict the risk of lymph node metastasis. In addition, high TLR4 expression was significantly associated with larger tumor size (≥2 cm) and later clinical stage, suggesting that high TLR4 expression may be the cause of postoperative metastasis and cancer recurrence. The expression of certain receptors in breast cancer is associated with multiple factors, of which TLR4 expression may be one, however, the findings are still controversial. In the present meta-analysis, the expression of TLR4 in the PR negative group was significantly higher than that in the PR positive group, while there was no statistically significant difference between the ER negative group and the ER positive group, and between the HER-2 negative group and the HER-2 positive group. TLR4 expression and survival prognostic indicators (OS or DFS) of breast cancer patients have also been reported. We conducted a meta-analysis of three studies. Although two had the same first author and the selection time of samples overlapped, the samples came from different databases, so the study results met the inclusion criteria. TLR4 expression was an independent prognostic indicator of DFS in breast cancer patients, but not related to OS. Due to the small number of included literatures, more studies with larger sample sizes are needed in the future to further confirm the conclusions.

This Meta-analysis mainly has the following shortcomings: First, most of the studies we included were conducted or published in China, and the results may only apply to Chinese or Asian populations, and publication bias is almost inevitable; Secondly, the experimental methods used in most studies were immunohistochemical analysis, and the results may be affected by the antibodies used, antibody concentration, storage time, fixation method of paraffin-embedded tissue, and critical values. Finally, we only selected a limited number of studies for the meta-analysis, and more high-quality studies are needed in the future to support the results.

## 5 Conclusion

Despite some limitations in this paper, the results are still significant. From our main analysis results, high TLR4 expression is associated with lymph node metastasis, larger tumor size (≥2 cm), later clinical stage, negative PR expression and shorter DFS, suggesting poor prognosis in breast cancer patients. Future prospective studies with long-term follow-up are needed to further validate the correlation between TLR 4 expression level and disease prognosis.

## Author contributions

JW: Data curation, Writing – original draft. JZ: Writing – original draft. XW: Writing – review & editing. XY: Writing – review & editing. XQ: Writing – review & editing. YW: Supervision, Writing – review & editing.
